# Emphysematous Cholecystitis Secondary to Fusobacterium nucleatum

**DOI:** 10.7759/cureus.15660

**Published:** 2021-06-15

**Authors:** Anuj Kunadia, Michael B Leong, Karthikram Komanduri, Randa Abdelmasih, Aneta Tarasiuk-Rusek

**Affiliations:** 1 Internal Medicine, University of Central Florida College of Medicine, Orlando, USA; 2 Internal Medicine, University of Central Florida, Orlando, USA; 3 Internal Medicine, Ocala Regional Medical Center, University of Central Florida College of Medicine, Ocala, USA; 4 Internal Medicine, Ocala Regional Medical Center, University of Central Florida, Ocala, USA; 5 Infectious Disease, Ocala Regional Medical Center, University of Central Florida College of Medicine, Ocala, USA

**Keywords:** emphysematous cholecystitis, fusobacterium nucleatum emphysematous cholecystitis, culture negative, anaerobic, fusobacterium nucelatum

## Abstract

*Fusobacterium nucleatum* may be implicated in cases of emphysematous cholecystitis (EC) and carries a high mortality risk, especially in individuals with heart disease, renal insufficiency, and underlying malignancy. *Fusobacterium* infections are rarely detected in the setting of cholecystitis possibly due to the difficulty with properly culturing the bacteria. We describe a case of a patient with EC in whom blood cultures were positive for growth of *F. nucleatum* in one of two samples. The patient was treated with empiric antibiotic therapy consisting of metronidazole and cefepime. In patients with EC and negative cultures, it is possible that they may have an undetected infection with fusobacteria, which carries a high mortality risk. As such, clinicians should maintain a high degree of suspicion of obligate anaerobic infection in patients who have negative blood culture for growth in the setting of EC and consider continuation of adequate antimicrobial coverage.

## Introduction

Emphysematous cholecystitis (EC) is an acute variant of cholecystitis, characterized by air in the gallbladder lumen, wall, or surrounding tissues without an abnormal communication with the gastrointestinal tract [1]. Acute cholecystitis is associated with inflammation of the gallbladder, with at least 90% of cases arising from cholelithiasis. The rare emphysematous variant is associated with less than 3% of cholecystitis cases with a mortality rate of 15%-20% in comparison to the 1.4% mortality in patients with cholecystitis. It should be noted that patients with EC may also have concurrent gallstones [2,3]. The underlying etiology is thought to be infection resulting in gangrene and subsequent necrosis of the gallbladder. EC is most commonly seen in males over 50 years old. Other associated medical conditions include cardiovascular disease, diabetes, and neoplasms [4]. 

Patients usually present with right upper quadrant pain, fever, nausea, vomiting, and occasionally positive Murphy’s sign [5,6]. Ultrasound (US) is the best initial imaging modality for detection, although the ability to visualize EC varies based on the amount of air in the gallbladder. The most sensitive imaging technique is CT. Diagnosis of EC is based on the clinical presentation and imaging [5,7]. EC is approached as a life-threatening condition, so patients should be administered antibiotics and undergo a cholecystectomy as soon as possible.

Secondary bacterial infections in acute cholecystitis are commonly due to the enteric organisms *Escherichia coli*, *Klebsiella*, *Streptococcus faecalis*, and *Enterococci* [8,9]. *Clostridium perfringens* is uniquely seen in patients with EC [10].

Our patient was found to have bacteremia secondary to *Fusobacterium nucleatum* in the setting of EC. Fusobacteria are obligate anaerobic bacteria commonly found in the oropharyngeal and gastrointestinal tracts [11,12]. Infection is usually associated with colorectal carcinoma and immunocompromised individuals [13]. Fusobacteria are usually sensitive, with low rates of resistance, to penicillins, metronidazole, and clindamycin [12,14].

## Case presentation

**Figure 1:**
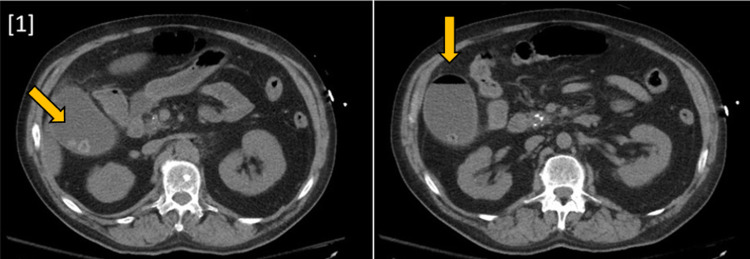
CT abdomen and pelvis without contrast showing a distended gallbladder with air-fluid collection and multiple stones as well as a peri-cholecystic fluid reaction.

**Figure 2:**
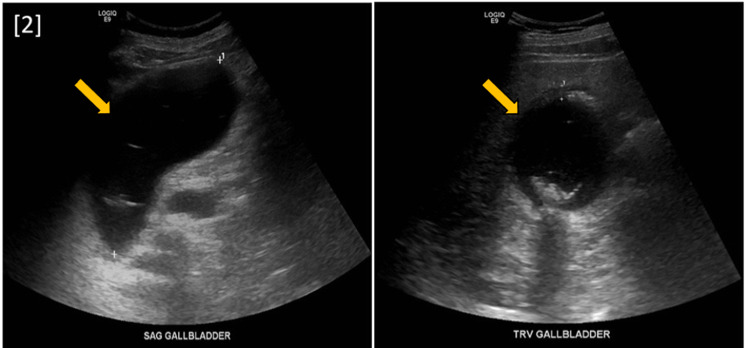
Right upper quadrant ultrasound (sagittal and transverse sections) showing moderately distended gallbladder with sludge and stones. Additionally, air present in the fundus is highly suggestive of emphysematous cholecystitis.

**Figure 3:**
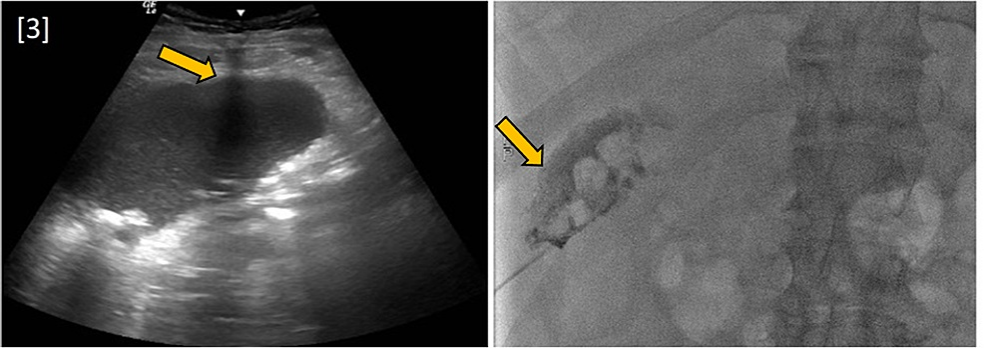
Fluoroscopic image-guided percutaneous cholecystostomy tube placement.

A 63-year-old male with a history of extensive alcohol and drug use, pancreatitis, hypertension, and gout was brought to the emergency department by emergency medical services due to a few days of stupor. Physical examination was significant for blood pressure of 167/87 and pain in the right upper quadrant. Labs demonstrated anion gap metabolic acidosis, rhabdomyolysis, hyponatremia, elevated liver enzymes, and acute kidney injury with severe uremia. Non-contrast CT of the abdomen and pelvis showed a distended gallbladder with an air-fluid level, confirming the diagnosis of EC (Figure 1). These findings were consistent with those found in the initial US (Figure 2). The patient received an emergency percutaneous cholecystostomy tube (Figure 3) and was started on empiric antibiotic therapy consisting of metronidazole and cefepime. Later in the course of admission, one of two blood cultures was positive for growth of *F. nucleatum*.

## Discussion

The prevalence of *Fusobacterium* in cholecystitis is likely underestimated due to the low incidence of positive blood cultures and the methods of obtaining fluid samples. *Fusobacterium nucleatum* is a known cause of odontogenic infections, pleural empyemas, and brain abscesses [15]. *Fusobacterium nucleatum* infections can also precipitate Lemierre Syndrome, a thrombophlebitis of the internal jugular vein [16]. However, *Fusobacterium* bacteremia is an uncommon infection, which accounts for approximately 0.9% of patients with bacteremia and carries an overall mortality rate of 40.7% even when patients receive appropriate antibiotic treatment. Heart failure, renal insufficiency, and malignancy are independent risk factors for mortality [17]. The low prevalence of *Fusobacterium* blood cultures is partially due to the fact that the organism is an obligate anaerobe. One study showed that appendicitis caused by anaerobic organisms only resulted in a positive blood culture 50% of the time [18]. 

Another source of low prevalence of EC secondary to *Fusobacterium* may be the method through which culture specimens are obtained. The specimens must be obtained and transported in an anaerobic environment. There are multiple documented cases of patients infected with *F. nucleatum* in which specimen cultures were negative for growth. In these cases, the infection was only detected when 16S rRNA gene polymerase chain reaction of the sample was utilized [19].

Cholecystostomy tubes are an easy way to access fluid from the gallbladder, especially in patients who are not candidates for cholecystectomy. However, the tube exposes the fluid to air, which can kill obligate anaerobic bacteria. Directly collecting sample fluid in an anaerobic environment from the gallbladder may be more likely to grow the bacteria responsible for EC. Identification of *Fusobacterium* may not alter management in the setting of acute cholecystitis as it is sensitive to current recommended antibiotic therapy [20]. However, clinicians should have suspicion for anaerobic etiology for EC in the setting of negative fluid and blood cultures, and continuation of antibiotic treatment should be considered to cover for *Fusobacterium* and other obligate anaerobic microorganisms that may be difficult to culture.

## Conclusions

Fusobacteria may be implicated in cases of EC. Bacteremia with this organism carries a high mortality risk, especially in individuals with heart disease, renal insufficiency, and underlying malignancy. *Fusobacterium* infections are rarely detected in the setting of cholecystitis and this may be due to the difficulty culturing the bacteria with current techniques. Despite negative cultures, patients with EC may have infection with fusobacteria, which carries a high mortality risk. As such, clinicians should maintain a high degree of suspicion of obligate anaerobic infection in patients who have negative blood culture for growth in the setting of EC and consider continuation of adequate antimicrobial coverage.
